# Author Correction: A gate tunable transmon qubit in planar Ge

**DOI:** 10.1038/s41467-024-53910-1

**Published:** 2024-10-31

**Authors:** Oliver Sagi, Alessandro Crippa, Marco Valentini, Marian Janik, Levon Baghumyan, Giorgio Fabris, Lucky Kapoor, Farid Hassani, Johannes Fink, Stefano Calcaterra, Daniel Chrastina, Giovanni Isella, Georgios Katsaros

**Affiliations:** 1 Institute of Science and Technology Austria, Klosterneuburg, Austria; 2 NEST, Istituto Nanoscienze-CNR and Scuola Normale Superiore, Pisa, Italy; 3 L-NESS, Physics Department, Politecnico di Milano, Como, Italy

**Keywords:** Qubits, Two-dimensional materials

Correction to: *Nature Communications* 10.1038/s41467-024-50763-6, published online 30 July 2024

The original version of this Article omitted the following from the Acknowledgements:

We acknowledge financial support from the Horizon Europe Framework Program of the European Commission through the European Innovation Council Pathfinder grant no. 101115315 (QuKiT).

This has now been corrected in both the PDF and HTML versions of the Article.

