# Supplementary material for: A gate tunable transmon qubit in planar Ge
Source: Nat Commun. 2024 Jul 30;15:6400. doi: 10.1038/s41467-024-50763-6 (PMC11289319; doi:10.1038/s41467-024-50763-6)
Supplement: Supplementary file 1 — Supplementary Information [file 41467_2024_50763_MOESM1_ESM.pdf]

# Supplementary Information:
# A gate tunable transmon in planar Ge

## SUPPLEMENTARY NOTE 1: ADDITIONAL LOSS MECHANISMS

The measured $T_1$ times range from 75 to 20 ns. Here, we demonstrate that we are not limited by design constraints, i.e., decay to the resonator and the gate (drive) line. To estimate the Purcell limit, we extract the bare resonator properties as depicted in Supplementary Figure 1. The obtained Purcell limit at the smallest detuning reads [1]: $T_{\mathrm{Purcell}} = \left(\kappa\,(g/\Delta)^2\right)^{-1} \approx 400\,\mathrm{ns}$ ($\kappa \approx 2\pi \cdot 3.4\,\mathrm{MHz}$ and $\max(g/\Delta) \approx 0.34, |\Delta_{\mathrm{min}}| \approx 0.8\,\mathrm{GHz}$). The calculated Purcell limit is an order of magnitude higher than the measured $T_1$ times. Thus, it is not a limiting factor. The same analysis applies to the reference transmons. The coupling strength is derived from a power-dependent resonator spectroscopy measurement, shown in Supplementary Figure 7. Our analysis yields a coupling strength of approximately $g/2\pi \approx 94\,\mathrm{MHz}$ , a detuning of approximately $|\Delta| \approx 1.26\,\mathrm{GHz}$, and a linewidth of approximately $\kappa \approx 2.23 \cdot 2\pi\,\mathrm{MHz}$. These parameters result in a Purcell time ($T_{\mathrm{Purcell}}$) of approximately $12\,\mu\mathrm{s}$, which is two orders of magnitude higher than the measured $T_1$.

To estimate the relaxation to the drive line, we model the gatemon as an $LC$ resonator capacitively coupled to the unfiltered $Z_0 = 50\,\Omega$ designed (impedance of the drive line) transmission line, acting as a gate. The environmental $50\,\Omega$ impedance will dissipate the energy escaping the gatemon circuit. We estimate the $T_1$ limit following the formula in Refs. [2, 3]:

$$T_{\mathrm{drive}} \approx \frac{4C}{Z_0 C_{\mathrm{g}}^2 \omega^2} \approx 22\,\mu\mathrm{s}, \tag{1}$$

where $Z_0 \approx 50\,\Omega$ is the impedance of the drive line, $C \approx 85\,\mathrm{fF}$ is the capacitance of the gatemon circuit, $\omega \approx 2\pi \cdot 5\,\mathrm{GHz}$ is the resonant frequency and $C_{\mathrm{g}} \approx 0.57\,\mathrm{fF}$ is the capacitance between the gatemon circuit and the drive line.

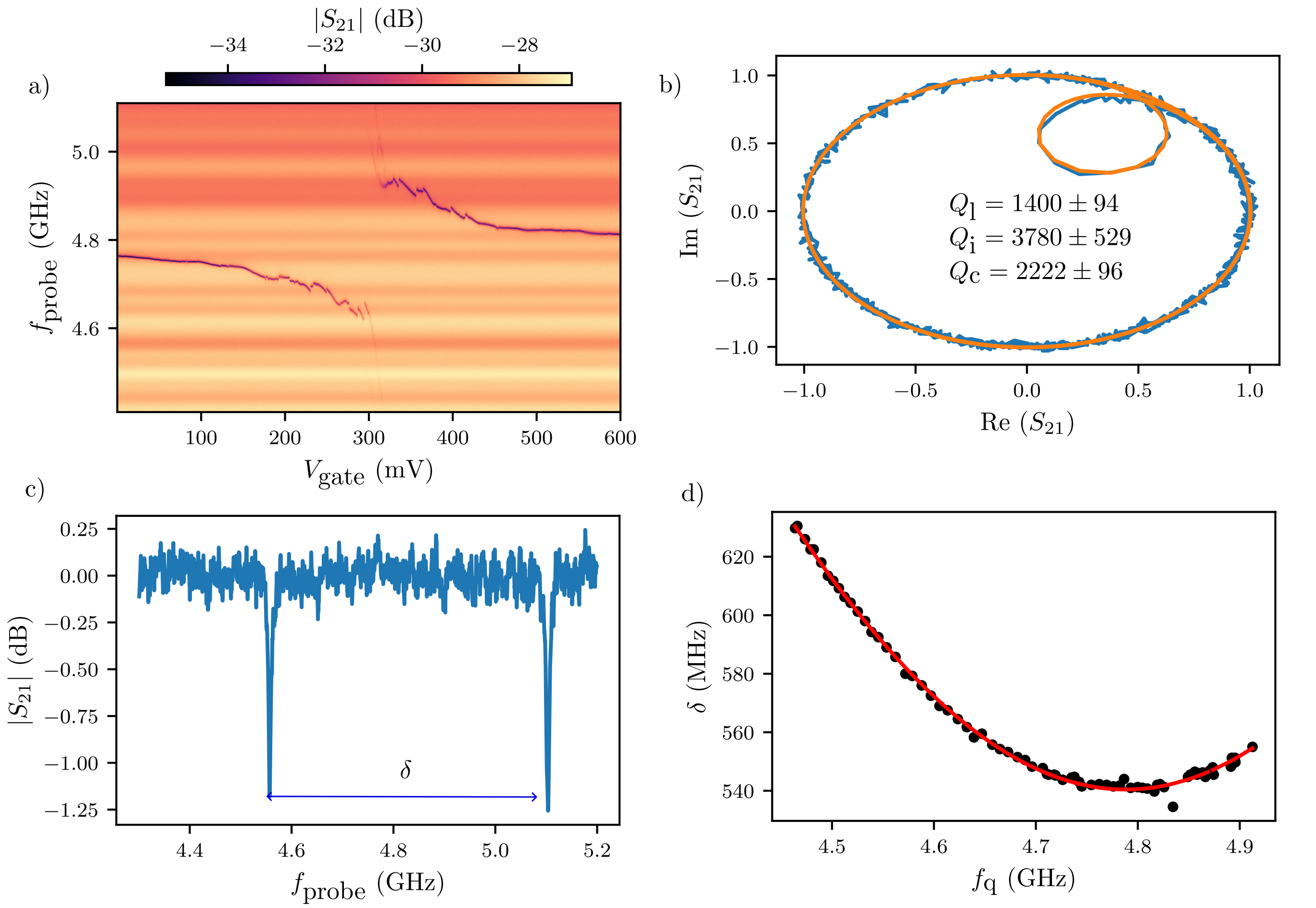


Supplementary Figure 1. Extended resonator spectroscopy data on the device presented in the main text. a) VNA sweep for a broader gate range without background correction at $n_{\text{ph}} \approx 1$ power level. b) Fit to extract the bare resonator properties taken at $V_{\text{gate}} = 700\,\text{mV}$ where the qubit is very far detuned from the resonator. We fit the complex $S_{21}$ parameter with the *resonator-tools* [4] package. c) Line-cut from Fig. 1e in the main text at $V_{\text{gate}} = 307\,\text{mV}$. We denote the splitting of the hybridized resonator-qubit states by $\delta$ as mentioned in the main text. d) Extracted splitting as a function of the qubit frequency. The solid red line is a fit according to Eq. 1 using $g$ as a free parameter.

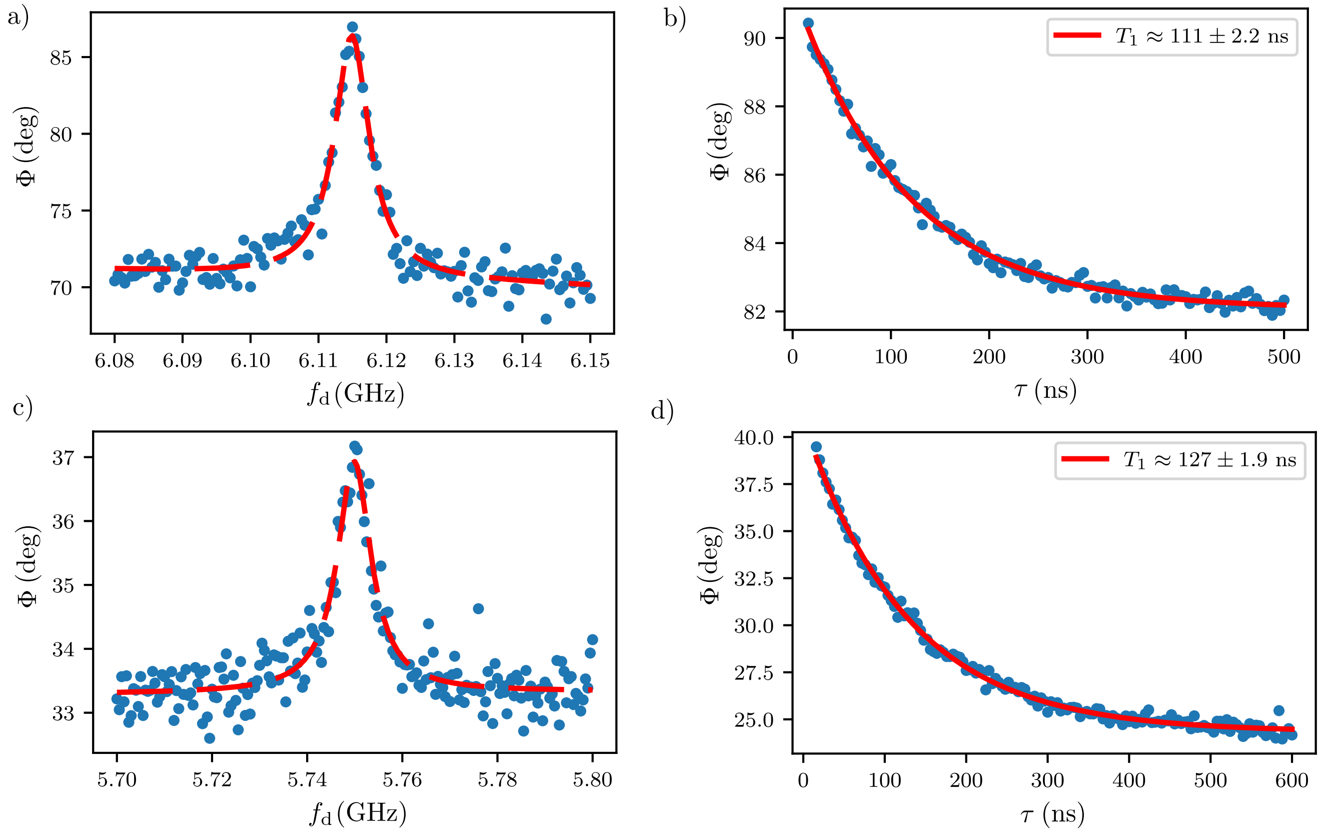


Supplementary Figure 2. Data from two additional Al transmons, one related to panels a) and b), the other to panels c) and d). The devices presented are identical to the one shown in Fig. 5, except for the junction size, which varies to get different qubit frequencies. a), c) Qubit spectroscopy with pulsed two-tone measurement. b), d) Energy relaxation measurements. The measured results are consistent with the ones presented in the main text.

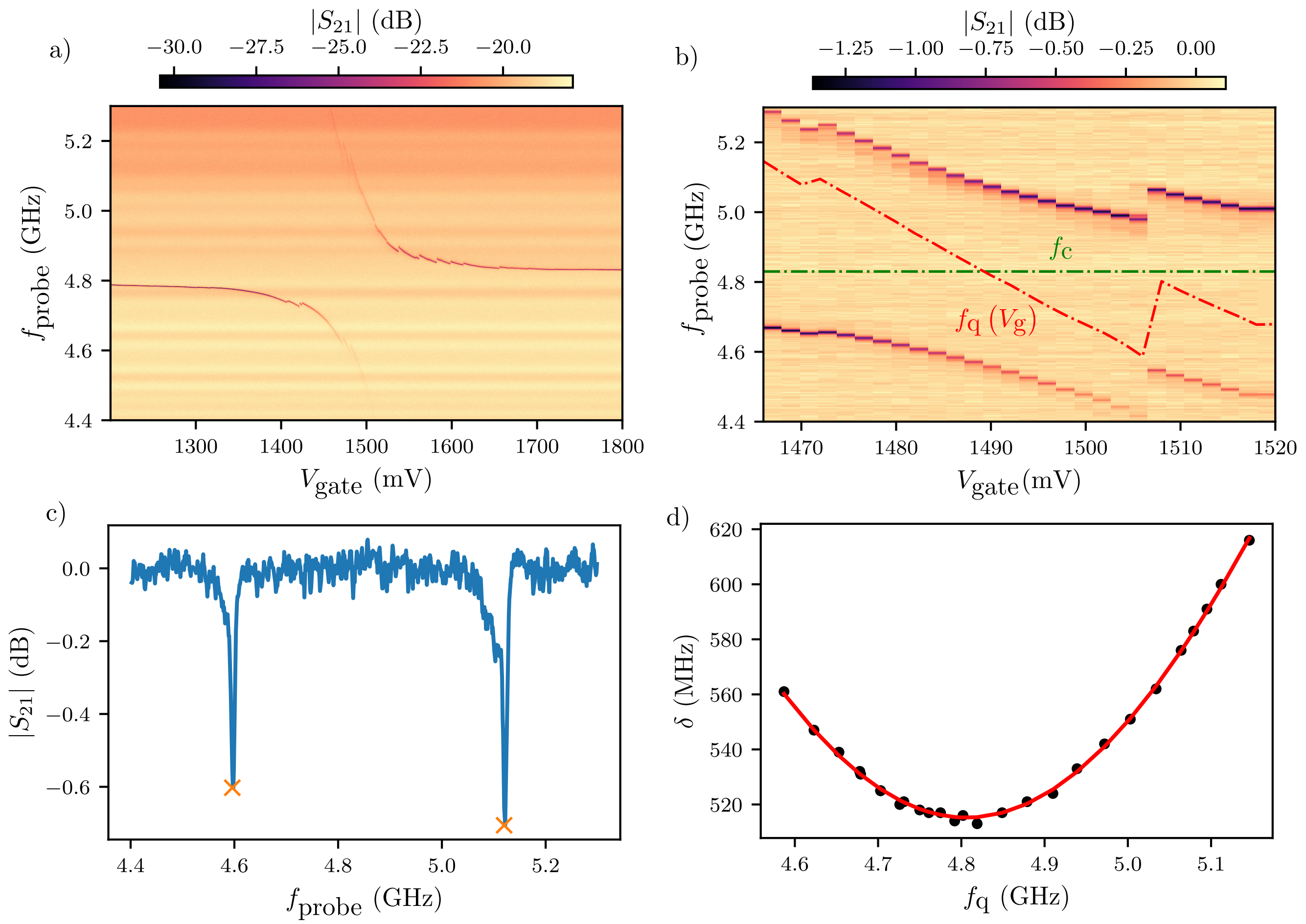


Supplementary Figure 3. Resonator spectroscopy on a second Ge gatemon device. Apart from the junction length, which is 50 nm shorter, the second device is nominally identical to the first one discussed in the main text. a) VNA sweep at low photon number. Since the junction is shorter compared to the sample of the main text, we needed to operate in a different gate voltage range to obtain the same critical current. b) Zoom-in of panel a). We observe two peaks due to qubit-resonator hybridization. c). One-dimensional line-cut at $V_{\text{gate}} = 1494\,\text{mV}$. d) Coupling strength extraction in the same way as demonstrated in Fig. 1. We extract a coupling value of $g/2\pi \approx 257\,\text{MHz}$, consistent with $g$ obtained in Fig. 1d.

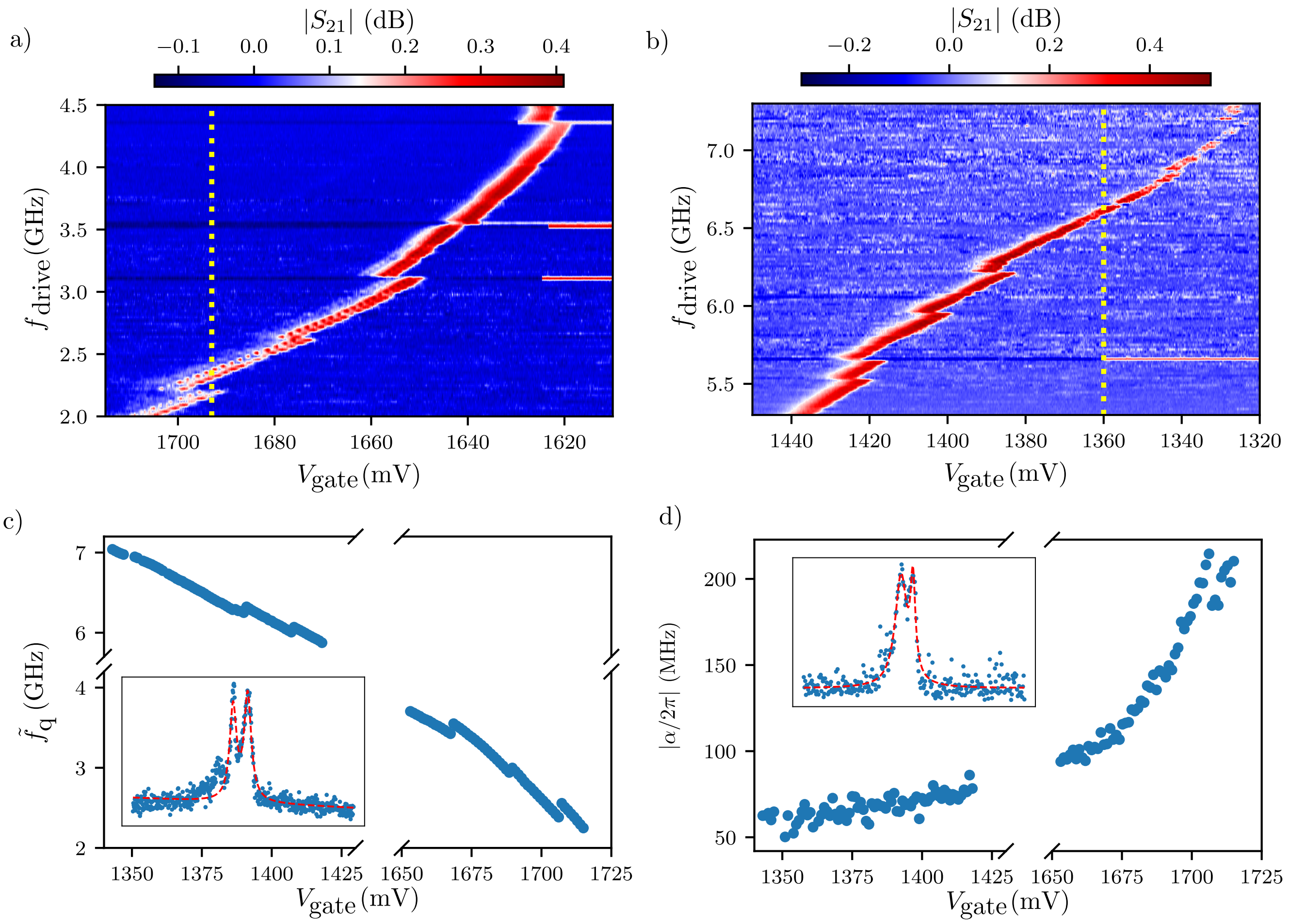


Supplementary Figure 4. Continuous-wave two-tone spectroscopy on a second gatemon device. The simplicity of continuous measurements allowed for quick characterization. a)-b) Raw two-tone spectroscopy data acquired with a VNA. We choose a high enough power to see the $|0\rangle \to |2\rangle$ transition via a two-photon process to obtain the anharmonicity with the same measurement. The qubit broadens due to photon shot noise as it moves closer to the resonator since the measurement and drive tone are applied simultaneously. We have subtracted the average of each column at every gate voltage. c) Extracted qubit frequencies versus gate voltage. The inset shows a linecut at $V_g = 1693\,\mathrm{mV}$ indicated by the yellow dashed line in panel a). We fit each trace with a double Lorentzian curve. The center of the second peak yields the qubit frequency. d) Extracted anharmonicity versus gate voltage. The inset shows a linecut at $V_{\rm gate} = 1360\,\mathrm{mV}$ indicated by the yellow dashed line in panel b). The distance between the peaks is the half of the anharmonicity.

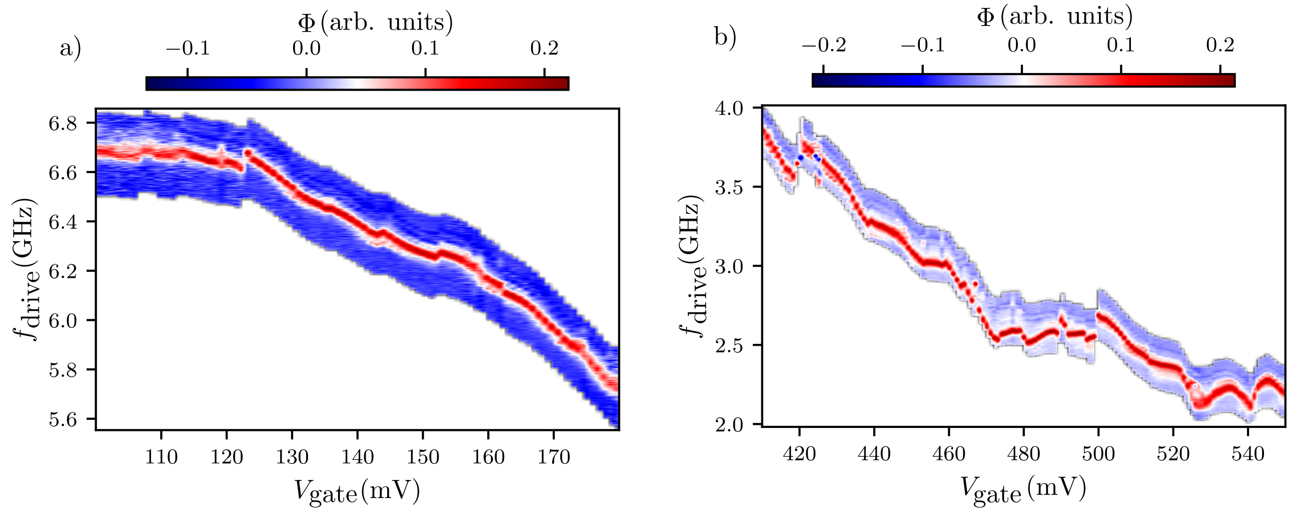


Supplementary Figure 5. Three-tone spectroscopy measurements used to extract the anharmonicity presented in Fig. 2d, with $f_{\mathrm{qubit}} > f_{\mathrm{r}}$ and $f_{\mathrm{qubit}} < f_{\mathrm{r}}$ shown in panel a) and b) respectively. The position of the measured peak yields the frequency of the $|1\rangle \rightarrow |2\rangle$ transition. At $V_{\mathrm{gate}} < 100\,\mathrm{mV}$, we observe two peaks in the two-tone spectroscopy measurement. Thus, we have left that region out in that measurement.

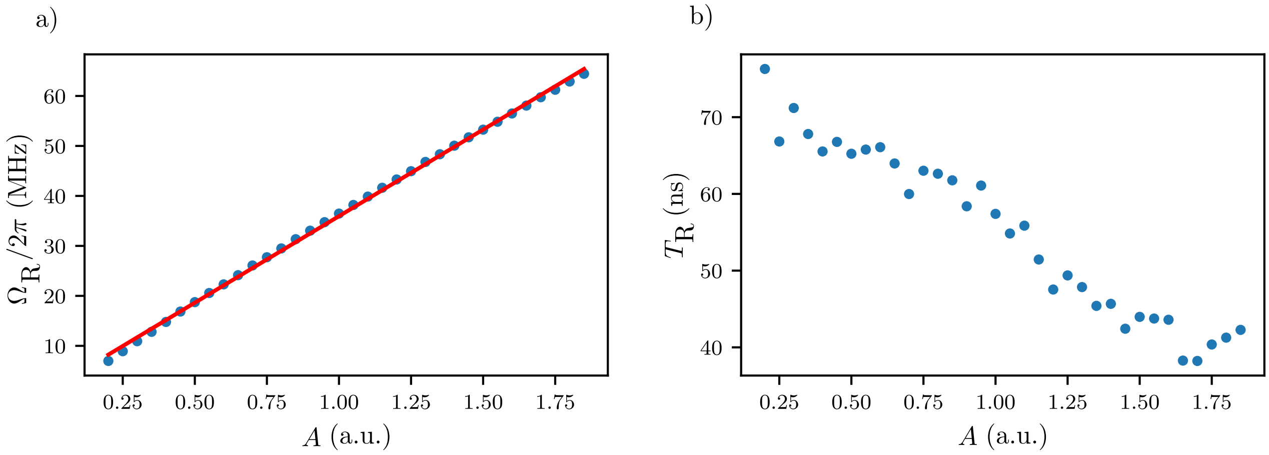


Supplementary Figure 6. Rabi frequency (panel a) and Rabi times (panel b) as a function of amplitude extracted from Fig. 3 from the main text. At each amplitude, we fitted the trace with the following expression: $A\cos(\Omega_{\mathrm{R}}t + \varphi)\exp(-t/T_{\mathrm{R}}) + B$.

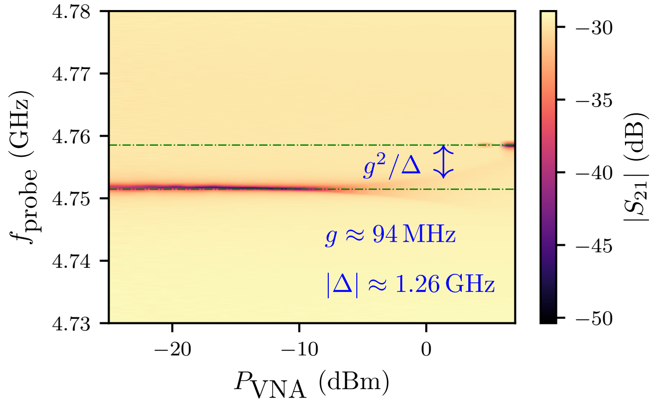


Supplementary Figure 7. Normalized feed line transmission of the reference transmon as a function of readout power. We observe a Lamb shift $\chi_0 = g^2/\Delta \approx 7$ MHz as a signature of coupling to a non-harmonic system. We extract the linewidth $\kappa$ of the resonator at $P_{\rm VNA} = -25$ dBm.

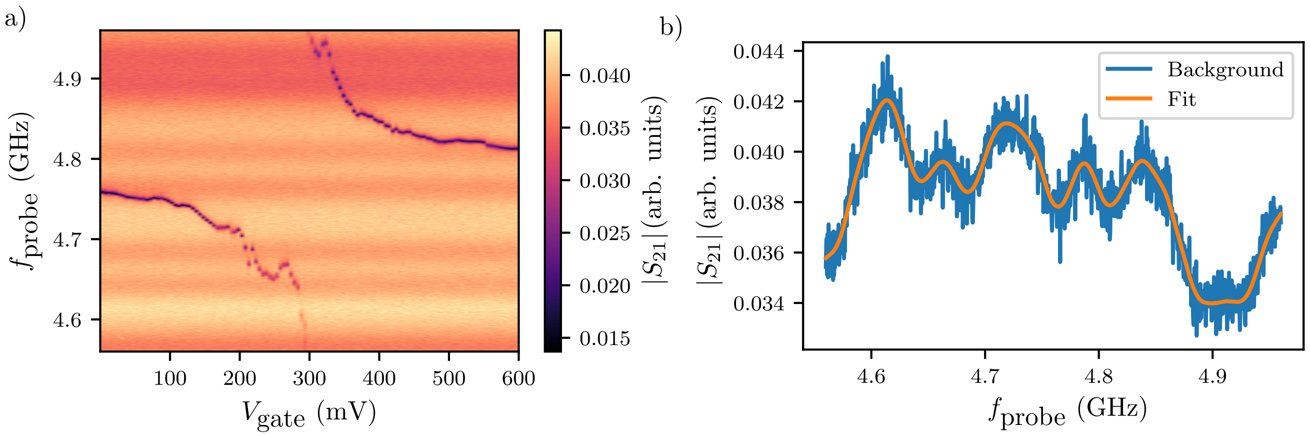


Supplementary Figure 8. Background correction. a) First, we reconstruct the background by concatenating the data from the purple rectangles. b) We fit the extracted background with a spline using the *resonator-tools* package. We corrected the data in Fig. 1e with the fitted background.

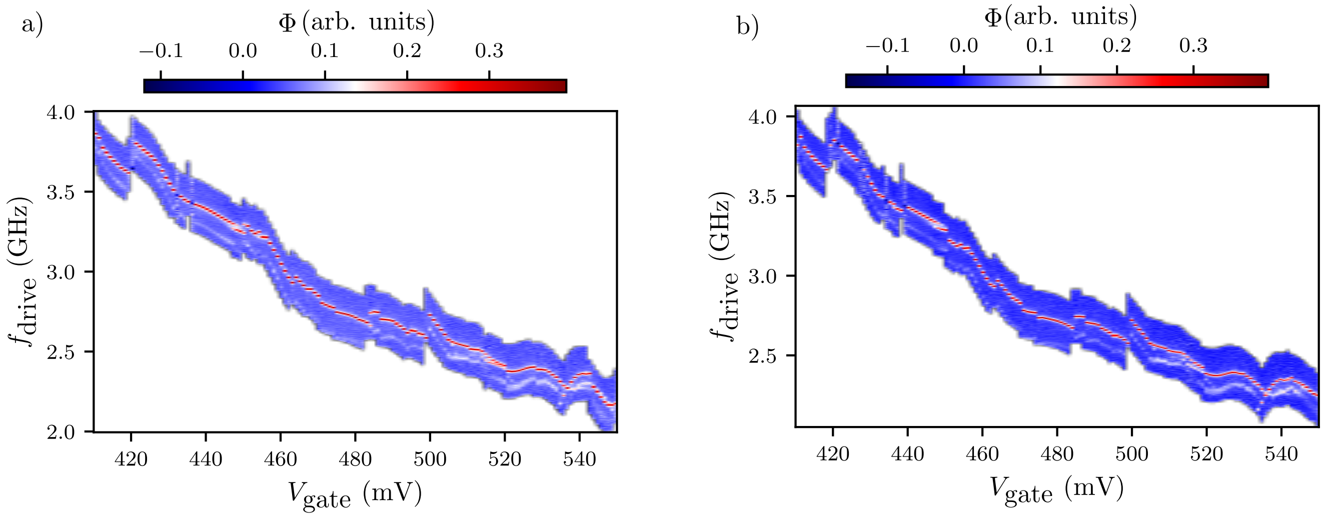


Supplementary Figure 9. Qubit spectroscopy measurements performed on different days reproducing the data shown in the main text. We see that the majority of the charge jumps are reproducible.

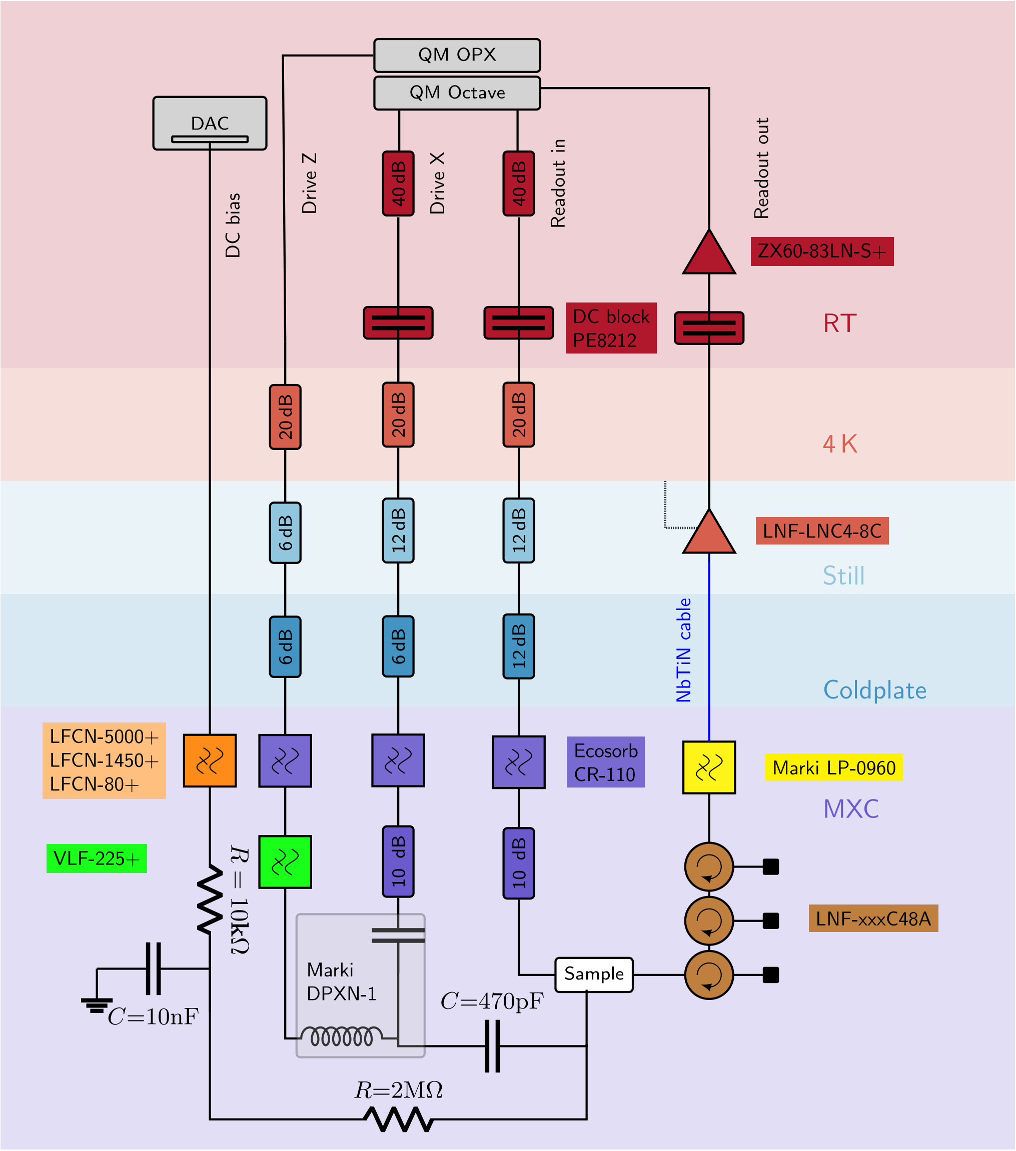


Supplementary Figure 10. Schematics of the fridge wiring and measurement setup. The *Readout in* and *Readout out* lines are connected to a VNA for continuous-wave measurements. For time-domain measurements, we have used Quantum Machines (QM) instruments. We have used *Drive X* line to send microwave (GHz) pulses, whereas the *Drive Z* line is intended for sending DC pulses. We combine the two lines at the MXC. The *Drive Z* line is not used in this experiment.

## SUPPLEMENTARY REFERENCES


[1] A. A. Houck, J. A. Schreier, B. R. Johnson, J. M. Chow, J. Koch, J. M. Gambetta, D. I. Schuster, L. Frunzio, M. H. Devoret, S. M. Girvin, and R. J. Schoelkopf, Controlling the spontaneous emission of a superconducting transmon qubit, Phys. Rev. Lett. **101**, 080502 (2008).

[2] S. Burkhard, Optimization of transmon design for longer coherence time (2012).

[3] J. Koch, T. M. Yu, J. Gambetta, A. A. Houck, D. I. Schuster, J. Majer, A. Blais, M. H. Devoret, S. M. Girvin, and R. J. Schoelkopf, Charge-insensitive qubit design derived from the cooper pair box, Phys. Rev. A **76**, 042319 (2007).

[4] S. Probst, F. B. Song, P. A. Bushev, A. V. Ustinov, and M. Weides, Efficient and robust analysis of complex scattering data under noise in microwave resonators, Review of Scientific Instruments **86**, 024706 (2015).
